# Preoperative Noninvasive Radiomics Approach Predicts Tumor Consistency in Patients With Acromegaly: Development and Multicenter Prospective Validation

**DOI:** 10.3389/fendo.2019.00403

**Published:** 2019-06-28

**Authors:** Yanghua Fan, Min Hua, Anna Mou, Miaojing Wu, Xiaohai Liu, Xinjie Bao, Renzhi Wang, Ming Feng

**Affiliations:** ^1^Department of Neurosurgery, Peking Union Medical College Hospital, Chinese Academy of Medical Sciences and Peking Union Medical College, Beijing, China; ^2^School of Electrical Engineering and Automation, East China Jiaotong University, Nanchang, China; ^3^Department of Radiology, Sichuan Provincial People's Hospital, Sichuan Academy of Medical Sciences, Chengdu, China; ^4^Department of Neurosurgery, The Second Affiliated Hospital of Nanchang University, Nanchang, China

**Keywords:** acromegaly, tumor consistency, magnetic resonance imaging, radiomics, ROC

## Abstract

**Background:** Prediction of tumor consistency before surgery is of vital importance to determine individualized therapeutic schemes for patients with acromegaly. The present study was performed to noninvasively predict tumor consistency based on magnetic resonance imaging and radiomics analysis.

**Methods:** In total, 158 patients with acromegaly were randomized into the primary cohort (*n* = 100) and validation cohort (*n* = 58). The consistency of the tumor was classified as soft or firm according to the neurosurgeon's evaluation. The critical radiomics features were determined using the elastic net feature selection algorithm, and the radiomics signature was constructed. The most valuable clinical characteristics were then selected based on the multivariable logistic regression analysis. Next, a radiomics model was developed using the radiomics signature and clinical characteristics, and 30 patients with acromegaly were recruited for multicenter validation of the radiomics model. The model's performance was evaluated based on the receiver operating characteristic (ROC) curve, area under the ROC curve (AUC), accuracy, and other associated classification measures. Its calibration, discriminating capacity, and clinical usefulness were also evaluated.

**Results:** The radiomics signature established according to four radiomics features screened in the primary cohort exhibited excellent discriminatory capacity in the validation cohort. The radiomics model, which incorporated both the radiomics signature and Knosp grade, displayed favorable discriminatory capacity and calibration, and the AUC was 0.83 (95% confidence interval, 0.81–0.85) and 0.81 (95% confidence interval, 0.78–0.83) in the primary and validation cohorts, respectively. Furthermore, compared with the clinical characteristics, the as-constructed radiomics model is more effective in prediction of the tumor consistency in patients with acromegaly. Moreover, the multicenter validation and decision curve analysis suggested that the radiomics model was clinically useful.

**Conclusions:** This radiomics model can assist neurosurgeons in predicting tumor consistency in patients with acromegaly before surgery and facilitates the determination of individualized therapeutic schemes.

## Introduction

Pituitary adenoma (PA), one of the most frequently seen benign pituitary gland neoplasms, constitutes about 10–25% of all primary intracranial tumors (1, 2); moreover, the prevalence of PA is showing an increasing trend (3). Acromegaly [overproduction of growth hormone (GH) by the PA in adult patients] is associated with numerous complications (4). Typically, the increased GH level will induce cardiovascular and cerebrovascular diseases, and acromegaly will increase the mortality by ≥2-fold compared with that among normal individuals (5, 6).

The Clinical Practice Guideline recommends surgical resection through the transsphenoidal approach, particularly the endoscopic transsphenoidal procedure, as the preferred first-line treatment for acromegaly (7, 8). Nonetheless, tumor consistency remains a major factor that affects the surgical resection rate because of the complicated anatomic structure in the sella region and the limited operative field of view, which is particularly true for macroadenomas (frequently seen among patients with acromegaly) (9, 10). Soft tumors can be easily curetted by means of suctioning. However, about 5–15% PAs are of the firm type (11); these tumors are more difficult to excise, and a two-stage operation is usually needed. Therefore, for firm PAs, surgeons may need to develop a more detailed strategy for the transsphenoidal procedure or directly choose craniotomy to avoid complications such as damage to the normal gland (which may lead to hypopituitarism) and interruption of the arachnoid membrane (which may result in cerebrospinal fluid fistulas and major cerebrovascular lesions). It is of vital importance to develop a noninvasive preoperative technique to enable precise prediction of the tumor consistency and thus to achieve a successful operative outcome and to establish an individualized therapeutic scheme, such as drug treatment (12).

A few studies have been performed to predict the consistency of PA through magnetic resonance (MR) imaging (11, 13). However, this technique is not always successful, and previous studies have been limited by low predictive accuracy, small sample sizes, lack of validation, and complicated imaging sequences. Radiomics has recently emerged as a potent approach for noninvasive high-throughput mining of tumor characteristics (14, 15). Neuro-oncologic radiomics studies can potentially mine hidden data that cannot be mined through a single-parameter approach; such studies can also enhance the accuracy of diagnosis, prognosis, and prediction in patients with brain tumors (16–19). Nonetheless, no report has focused on radiomics signatures to predict the tumor consistency of PA, particularly in patients with acromegaly.

Therefore, the hypothesis of the present study was that the high-dimensional radiomics characteristics acquired, based on conventional MR imaging sequences, can enhance the accuracy of preoperative prediction of the tumor consistency. Efforts were made to validate the constructed MR radiomics model through a completely independent multicenter validation set, thus offering robustness among different image acquisition protocols. Consequently, the current study aimed to construct a radiomics model that incorporates both the radiomics signature and clinical characteristics for preoperative prediction of the tumor consistency in patients with acromegaly.

## Materials and Methods

### Patients

From July 2012 to June 2018, 158 patients with acromegaly were included in the current analysis. The following four diagnostic criteria for acromegaly were used: clinical features of acromegaly in an adult patient (8), detection of a PA by pituitary MR imaging, meeting of the endocrine diagnostic criteria for acromegaly (8) [elevated insulin-like growth factor 1 level (20), random GH level of >1 ng/ml, and nadir GH level of >0.4 ng/ml after the oral glucose tolerance test], and confirmation of PA by postoperative pathological analysis.

The patients' data and personal information were anonymized before analysis. All patients were then randomized into the primary cohort (*n* = 100), which was used for model construction, and the validation cohort (*n* = 58), which was used for model internal validation. Finally, 30 patients with acromegaly were enrolled to prospectively validate the model, including 15 from Peking Union Medical College Hospital, 10 from Sichuan Provincial People's Hospital, and 5 from the Second Affiliated Hospital of Nanchang University. The Ethical Review Committee of Peking Union Medical College Hospital approved the study protocol.

The inclusion criteria for this study was as follows: the presence of acromegaly and performance of initial neurosurgery for PA removal; greatest tumor diameter of >2 cm or grade III to IV tumor (21); high-quality preoperative pituitary MR imaging and surgical video; no history of medical treatment, radiotherapy, or surgery for PA; and complete clinical and surgical data.

**Figure 1:**
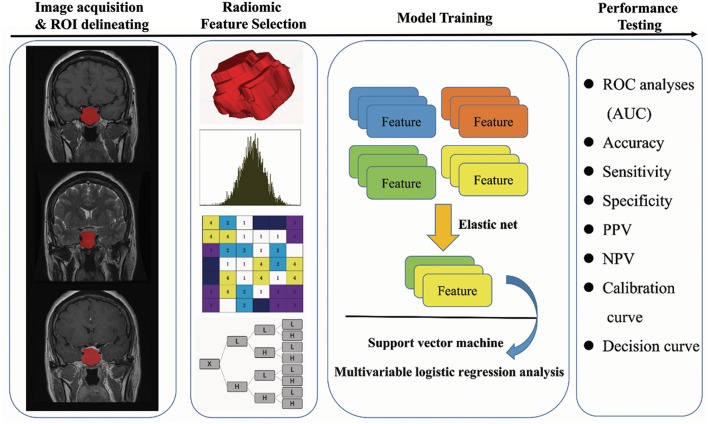
Flow chart of the present study. (I) Original MR image acquisition and ROI segmentation. (II) Radiomics features were extracted from the ROIs, including first-order features, textural features, wavelet features, and shape features. (III) Feature selection and model training. (IV) Performance testing of the model (ROC analysis, calibration curve analysis, decision curve analysis, and multicenter prospective validation). Soft and firm consistency were classified according to the neurosurgeon's evaluation: a tumor that could be suctioned out using an aspirator was considered soft, while a tumor that could not be suctioned out was considered firm.

The following preoperative clinical features were collected: age, sex, insulin-like growth factor 1 level, random GH level, nadir GH level and GH inhibition ratio after a glucose load, tumor volume, and bilateral Knosp classification (21). After surgery, the tumor consistency was classified as soft or firm according to the classification method described by Bahuleyan et al. (22); specifically, a tumor that could be suctioned out using an aspirator was considered soft, while a tumor that could not be suctioned out was considered firm. A high-quality surgical video was viewed by two neurosurgical experts with >10 years of related experience to determine the tumor consistency. A flow chart of this study is presented in Figure 1.

### MR Imaging Protocol and Image Acquisition

All patients underwent pituitary MR imaging before surgery. The precise MR imaging protocol included T2-weighted imaging (T2WI), T1-weighted imaging (T1WI), and contrast-enhanced T1WI. MR imaging was performed in the head-first supine position using a 3.0-T magnetic resonance system (Discovery MR 750, GE Healthcare, Chicago, IL, USA). Specifically, the T2WI sequence acquisition parameters were as follows: repetition time/echo time of 4,200/103 ms, field of view of 200 × 200 mm, flip angle of 90°, acquisition matrix of 320 × 224, slice thickness of 4 mm, and spacing between slices (center to center of each slice) of 5 mm. Additionally, the T1WI sequence acquisition parameters were as follows: repetition time/echo time of 400/9 ms, field of view of 200 × 200 mm, flip angle of 90°, acquisition matrix of 288 × 192, slice thickness of 3 mm, and spacing between slices of 3.5 mm. The contrast-enhanced T1WI sequence acquisition parameters were the same as the T1WI sequence parameters. Contrast-enhanced T1WI was carried out immediately after rapid injection of a gadolinium-DTPA contrast agent (0.1 mmol/kg Gadovist; Bayer AG, Leverkusen, Germany). T2WI, T1WI, and contrast-enhanced T1WI in the coronal plane were utilized, and all images were collected based on the picture archiving and communication system of the hospital.

### Tumor Masking and Feature Extraction

A neuroradiologist with 7 years of experience in studying acromegaly was responsible for drawing the related regions of interest (ROIs) that delineated the tumor in each patient with acromegaly on the aforementioned MR images through ITK-SNAP software (University of Pennsylvania, www.itksnap.org). The as-drawn ROIs were confirmed manually by another expert neuroradiologist with 12 years of related experience in investigating acromegaly, and this neuroradiologist was blind to the operation records. Any disagreement between the two neuroradiologists was settled by mutual negotiation between them. The radiomics features were then collected based on these ROIs.

In total, 1,561 quantitative features were collected for every sequence using PyRadiomics (https://github.com/Radiomics/pyradiomics) (23). All radiomics features were normalized to a value of 0 to 1 and classified into four groups: first-order features (*n* = 180), shape and size features (*n* = 13), textural features (*n* = 680), and wavelet features (*n* = 688). The textural features included the gray-level co-occurrence matrix (GLCM), gray-level run-length matrix, and the gray-level size-zone matrix.

### Selection of Radiomics Features

High-dimensional information may be associated with a high level of redundant irrelevant information. This may result in overfitting, thus seriously reducing the learning algorithm performance. Therefore, it was necessary to carry out a feature selection process.

Features were selected by a three-stage process based on the results acquired from the primary cohort. Differences in the features between soft and firm cases in the primary cohort were examined by univariate analysis through the Wilcoxon rank sum test. The elastic net approach (24), which combined the least absolute shrinkage and selection operator algorithm with ridge regression, was adopted to select features with the highest representativeness. The final features were confirmed using the recursive feature elimination algorithm through 7-fold cross-validation.

### Establishment and Validation of the Radiomics Signature

The radiomics signature was established based on the radiomics features selected from the primary cohort through the support vector machine method. At the same time, differences in the signature distribution between soft and firm tumors were compared between the two cohorts using a violin plot. A receiver operating characteristic (ROC) (25) curve was drawn to display the predictive value of the as-selected radiomics signature.

### Construction and Validation of the Clinical and Radiomics Model

The Akaike information criterion (AIC) (26) was used to screen the clinical features, among which the most valuable ones were further screened through multivariable logistic regression analysis. To establish an individualized model for predicting the tumor consistency for both clinicians and patients, the constructed radiomics signature was used in combination with the selected clinical features, which were further screened according to the AIC, to produce a fusion radiomics model. A nomogram was obtained from the radiomics model.

ROC analyses and calculation of the associated classification measures [including the area under the curve (AUC), accuracy, sensitivity, specificity, positive predictive value, and negative predictive value] were performed to compare the discriminability of the clinical and radiomics model in the two cohorts. Calibration curves were then drawn and used in combination with the Hosmer–Lemeshow test to assess the similarities between the predicted and measured tumor consistency probabilities (27).

### Clinical Application and Multicenter Validation

The clinical usefulness of the radiomics model was assessed by means of a decision curve analysis, in which the net benefits were quantified under different threshold probabilities (28). Following construction of the radiomics model, the 30 patients with acromegaly from three hospitals were enrolled for multicenter validation of the model. The prediction accuracy was then evaluated through ROC analyses and associated classification measures.

### Statistical Analysis

Differences with a two-sided *p*-value of < 0.05 were deemed statistically significant. The statistical software R, version 3.4.1 (R Foundation for Statistical Computing, Vienna, Austria) was used by an experienced statistician to carry out the statistical analysis. The combined model was created using the “rms” package, the calibration plot was examined through the “hdnom” packages, and the decision curve analysis was conducted by the function “dca.R.”

## Results

### Clinical Characteristics

The clinical characteristics of all 158 patients with acromegaly (100 from the primary cohort and 58 from the validation cohort) enrolled in this study are shown in Table 1. In total, 38.0% (38/100) and 34.5% (20/58) patients in the primary cohort and validation cohort, respectively, developed firm tumors. No significant interclass differences were detected in age, sex, random GH level, nadir GH level and GH inhibition ratio following a glucose load, insulin-like growth factor 1 level, tumor volume, or Knosp classification between the two cohorts (*p* = 0.418–0.999). These results justify the use of these two sets as a primary cohort and validation cohort. Significant interclass differences in the tumor volume and Knosp classification were found in both cohorts, which might be ascribed to the correlations of these parameters with tumor consistency. Patient age was different between the soft and firm groups in the primary cohort only (Table 2).

**Table 1:** Characteristics of patients in the primary and validation cohorts.

**Characteristic**	**Whole cohort (*n* = 163)**	**Primary cohort (*n* = 100)**	**Validation cohort (*n* = 58)**	***P*-value**
Age (year)	37 (30–48)	37 (31–48)	38 (29–48)	0.418
**Gender**				
Female	95 (60.1%)	60 (60%)	35 (60.3%)	0.996
Male	63 (39.9)	40 (40%)	23 (30.7%)	
GH level (ng/ml)	16.4 (8.5–38.3)	16.0 (8.3–39.2)	19.7 (8.9–34.1)	0.976
Nadir GH level (ng/ml)	11.2 (4.9–27.1)	10.9 (4.7–31.4)	12.2 (5.3–24.1)	0.885
IGF-1 level (ng/ml)	817.04 ± 285.42	808.84 ± 290.06	831.17 ± 279.15	0.637
GH inhibition ratio^*^ (%)	31.0 (16.0–44.5)	30.7 (15.5–45.5)	31.4 (15.9–43.2)	0.885
Tumor volume	2.9 (1.0–7.4)	3.0 (1.1–7.3)	2.2 (1.0–2.2)	0.717
Knosp classification^**^				
Grade 0	5 (3.2%)	3	2	0.999
Grade 1	6 (3.8%)	4	2	
Grade 2	42 (26.6%)	27	15	
Grade 3	50 (%)	31	19	
Grade 4	55 (%)	35	20	
**Consistency**				
Soft	100 (63.3%)	62	38	0.658
Firm	58 (36.7)	38	20	

Categorical variables are presented as number (percentage). Continuous variables with a normal distribution are presented as mean ± standard deviation; otherwise, median and quartile are shown. The chi-square test or Fisher's exact test, as appropriate, was used to compare the differences in categorical variables, while the independent-samples t-test was used to compare the differences in continuous variables. The following preoperative data were obtained: age, sex, GH level, nadir GH level, GH inhibition ratio, insulin-like growth factor 1 level, tumor volume, and bilateral Knosp classification. An oral glucose tolerance test (OGTT) was carried out with 75 g of oral glucose and subsequent measurements of the glucose and GH levels every 30 min for 2 h. The nadir GH level is the lowest GH level in the OGTT.

* *The GH inhibition rate is the percentage of GH decrease after the OGTT [GH inhibition rate = (0 h GH level–nadir GH level)/0 h GH level]*.

** *In 1993, Knosp et al. (21) proposed a classification system for predicting the invasion of the cavernous sinus by a pituitary adenoma. The Knosp classification system was used to grade the tumors from 0 to 4 according to the transverse range of tumors related to the internal carotid artery on coronal MR images. The more lateral the pituitary adenoma grows and surrounds the internal carotid artery, the higher the grade*.

**Table 2:** Univariate analysis of clinical characteristics of patients and acromegaly in the primary cohort and validation cohort.

**Characteristic**	**Primary cohort (*****n*** **=** **100)**		***P*-value**	**Validation cohort (*****n*** **=** **58)**		***P*-value**
	**Soft**	**Firm**		**Soft**	**Firm**	
Age (year)	41.7 ± 11.0	36.0 ± 11.7	0.015	40.0 ± 12.1	36.1 ± 9.3	0.221
**Gender**						
Male	38	22	0.737	24	11	0.546
Female	24	16		14	9	
GH level (ng/ml)	13.8 (8.3–34.7)	18.8 (8.0–77.0)	0.196	20.8 (8.7–29.6)	16.3 (9.0–55.3)	0.545
Nadir GH level (ng/ml)	9.0 (4.5–26.5)	14.8 (6.1–54.8)	0.18	11.6 (5.0–23.5)	15.0 (7.0–31.1)	0.288
IGF-1 level (ng/ml)	804.2 ± 302.7	816.5 ± 271.9	0.839	801.5 ± 261.4	887.6 309.1	0.268
GH inhibition ratio (%)	33.2 ± 19.4	29.3 ± 22.4	0.357	33.4 19.9	26.4 17.6	0.195
Tumor volume	2.2 (0.8–5.5)	6.1 (1.7–10.5)	0.001	1.4 (0.7–5.2)	6.4 (3.0–10.9)	0.001
**Knosp classification**						
Grade 0	3	0	0.009	2	0	0.028
Grade 1	4	0		2	0	
Grade 2	20	7		13	2	
Grade 3	21	10		13	6	
Grade 4	14	21		8	12	

### Feature Screening and Establishment of the Radiomics Signature

**Figure 2:**
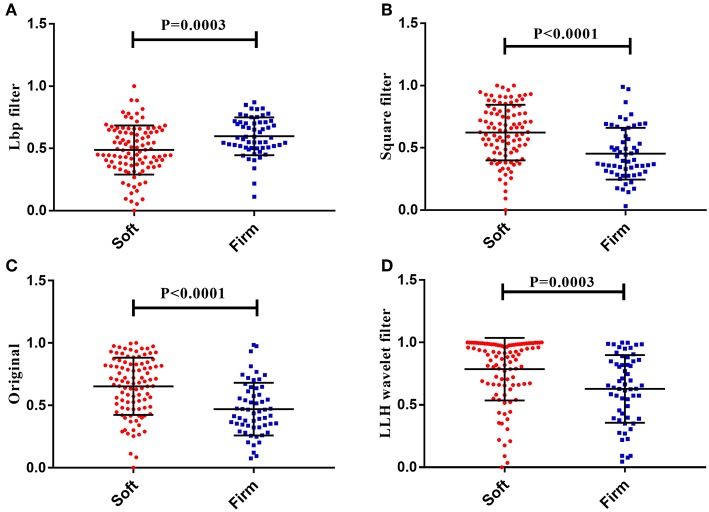
The four radiomics features showed significant differences between the soft and firm tumor groups. **(A)** Lbp filter gldm_DependenceVariance. **(B)** Square filter glcm_Imc2. **(C)** Original glcm_Imc2. **(D)** LLH wavelet filter glcm_Imc2.

**Figure 3:**
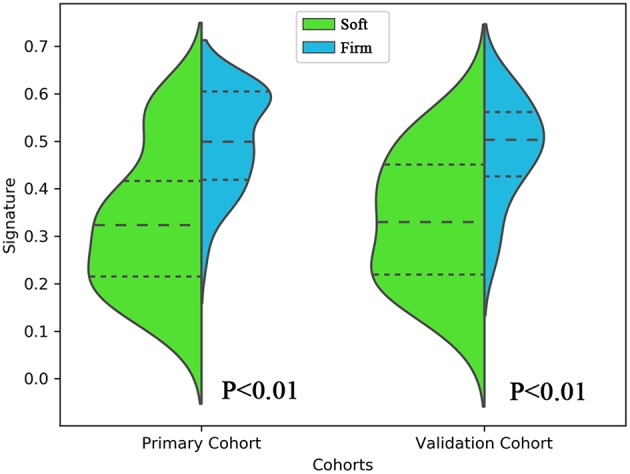
Violin plot comparing the distribution of the radiomics signatures of soft and firm tumors in the primary and validation cohorts. This plot is a combination of a boxplot and kernel density estimate. The signature distributions of each cohort were compared using independent-samples *t*-tests.

In total, 4,683 radiomics features were calculated in this study. Among these, the four key features were screened through the elastic net feature selection algorithm. The detailed information is presented in Table 3. The four selected radiomics features were statistically different between the two tumor consistencies (Figure 2). Additionally, differences in the radiomics signature distribution were statistically significant between the soft and firm tumors in both cohorts, as revealed by the violin plot (*p* < 0.01) (Figure 3).

**Table 3:** The four key radiomic features detail information.

**Class**	**Feature name**	**Feature type**	**Sequence**	**Soft**	**Firm**	***P*-value**
Lbp filter	gldm_DependenceVariance	Texture	T1WI	0.4876 ± 0.0196	0.5976 ± 0.0198	0.0003
Square filter	glcm_Imc2	Texture	CET1	0.6226 ± 0.0223	0.4534 ± 0.0272	<0.0001
Original	glcm_Imc2	Texture	CET1	0.6524 ± 0.0229	0.4700 ± 0.0277	<0.0001
LLH wavelet filter	glcm_Imc2	Wavelet	CET1	0.7864 ± 0.0251	0.6280 ± 0.0356	0.0003

*Lbp, local binary pattern; gldm, gray level dependence matrix; glcm, gray-level co-occurrence matrix; T1WI, T1-weighted imaging; CE-T1, contrast-enhanced T1-weighted imaging*.

**Figure 4:**
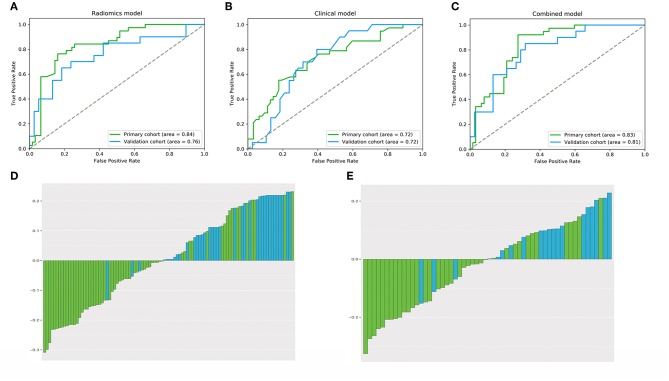
The performance of **(A–C)** ROC curves for the three models and **(D,E)** bar plots for the radiomics model in the primary and validation cohorts. The performance of the models was assessed using the AUC. **(A)** Radiomics signature. **(B)** Clinical model. **(C)** Radiomics model. **(D)** Primary cohort. **(E)** Validation cohort.

The radiomics signature based on the four selected radiomics features was then established according to the support vector machine model. The results suggested that the constructed radiomics signature had favorable performance in predicting tumor consistency, with an AUC of 0.84 [95% confidence interval (CI), 0.81–0.86] and 0.76 (95% CI, 0.73–0.79) in the primary and validation cohorts, respectively. The ROC curves derived from the two cohorts are shown in Figure 4A. All predictive indicators acquired based on the radiomics signature are shown in Table 4.

**Table 4:** Performance of radiomics signature, clinical features and radiomic model.

**Model**	**Performance**	**ACC (%)**	**AUC**	**SN (%)**	**SP (%)**	**PPV (%)**	**NPV (%)**
Radiomics signature	Primary cohort	81.00	0.84	76.30	82.30	72.50	85.00
	Validation cohort	75.90	0.76	65.00	81.60	65.00	81.60
Clinical features	Primary cohort	68.00	0.72	39.50	85.50	62.50	69.70
	Validation cohort	67.20	0.72	40.00	81.60	53.30	72.10
Nomogram	Primary cohort	80.00	0.83	92.10	72.60	67.30	93.80
	Validation cohort	78.00	0.81	85.00	70.40	58.60	89.70

*ACC, accuracy; AUC, area under curve; SEN, sensitivity; SPE, specificity*.

### Performance of the Clinical Radiomics Model

Using the AIC, age and the Knosp classification were chosen as the discriminatory factors to establish the clinical model. The AUCs were 0.72 (95% CI, 0.69–0.76) and 0.72 (95% CI, 0.69–0.75) in the primary and validation cohorts, respectively (Figure 4B). Additionally, the Knosp classification and radiomics signature were screened among various clinical features based on the AIC to establish the fusion radiomics model. The AUCs were 0.83 (95% CI, 0.81–0.85) and 0.81 (95% CI, 0.78–0.83) in the primary and validation cohorts, respectively, whereas the accuracy was 80.0 and 78.0% in the two cohorts, respectively (Figure 4C). Bar plots showing the prediction accuracy of the radiomics model for the two cohorts are shown in Figures 4D,E.

**Figure 5:**
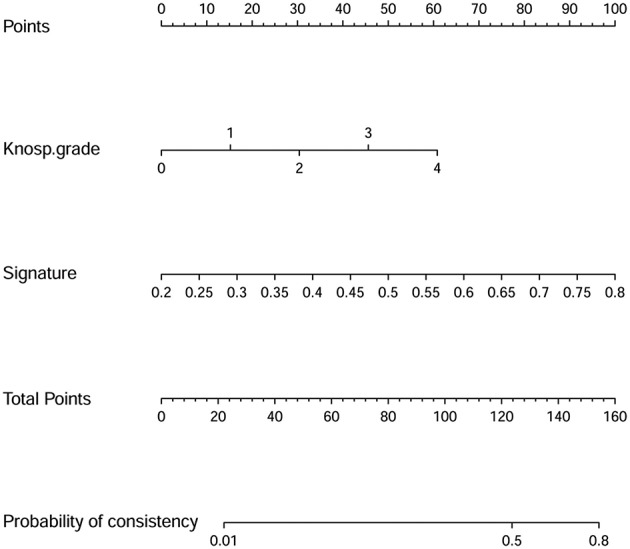
Nomogram derived from the radiomics model applied to the primary cohort. The radiomics model was incorporated with the Knosp grade and radiomics signature. This nomogram is used according to the patient's Knosp grade and radiomics signature value. The position of each variable is found on the corresponding axis (lines 2 and 3), and a vertical line is then drawn to the points axis (the first line) to obtain the corresponding score. As an example, one patient in this study with a Knosp grade of 3 had a score of 45.6, and his radiomics signature value was 0.28, which means a score of 13.0. Next, according to the sum of all scores, the position on the total points axis is identified, and a vertical line is drawn from the position to the last line to determine the tumor consistency possibilities. The above-mentioned patient's total risk score was 58.6, which corresponds to a 2.4% probability of a firm tumor. That is to say, using our nomogram, this patient's tumor consistency was predicted to be soft before surgery. Later, this patient's clinical and surgical data indicated that the tumor was indeed soft. Thus, the model accurately predicted this patient's tumor consistency.

**Figure 6:**
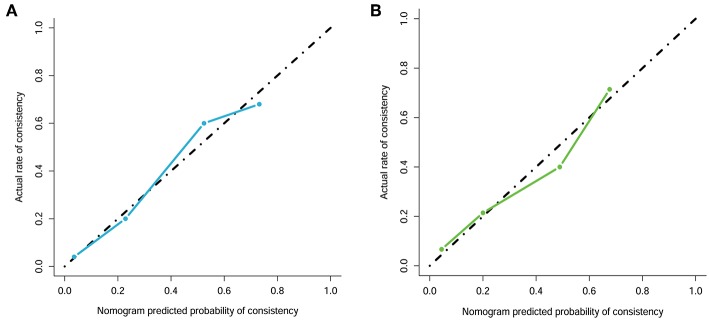
Calibration curve analysis for the radiomics model. **(A)** Primary cohort. **(B)** Validation cohort. Calibration curves depict the calibration of each model in terms of the agreement between the predicted and actual probability of the firm tumor rate. The Y axis represents the actual rate. The X axis represents the predicted probability. The diagonal black line represents perfect prediction by an ideal model. The blue and green line represents the performance of the radiomics model, of which a closer fit to the diagonal black line represents a better prediction.

Details of the predictive indicators for the clinical radiomics model are presented in Table 4. A nomogram was obtained based on the radiomics model (Figure 5). Additionally, the results of the DeLong test suggested that the performance of the radiomics model was superior to that of any independent clinical feature (*p* = 0.03). A calibration curve was also plotted based on the radiomics model to examine the tumor consistency and suggested excellent agreement between the observed and predicted results in both the primary cohort (*p* = 0.59) (Figure 6A) and validation cohort (*p* = 0.68) (Figure 6B). No statistical significance was detected by the Hosmer–Lemeshow test, suggesting the absence of a distinct departure from the perfect fit.

The radiomics model also achieved favorable discriminability and excellent calibration; it could more accurately predict the tumor consistency in patients with acromegaly than the use of clinical features only could.

### Clinical Usefulness of the Constructed Radiomics Model

**Figure 7:**
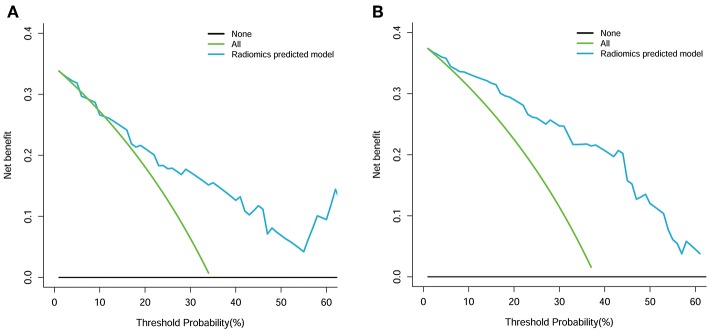
Decision curve analysis for the radiomics model. **(A)** Primary cohort. **(B)** Validation cohort. The Y axis measures the net benefit. The blue line represents the radiomics model. The green line represents the assumption that all patients had a firm tumor. The black line represents the assumption that no patients had a firm tumor.

The decision curve analysis of the radiomics model is presented in Figures 7A,B. The radiomics model clearly provided a net benefit over the two schemes, with a threshold probability of >0 and >12% for the primary and validation cohorts, respectively, suggesting the clinical usefulness of the radiomics model. The decision curve attained better performance for the constructed radiomics model with regard to clinical application.

**Figure 8:**
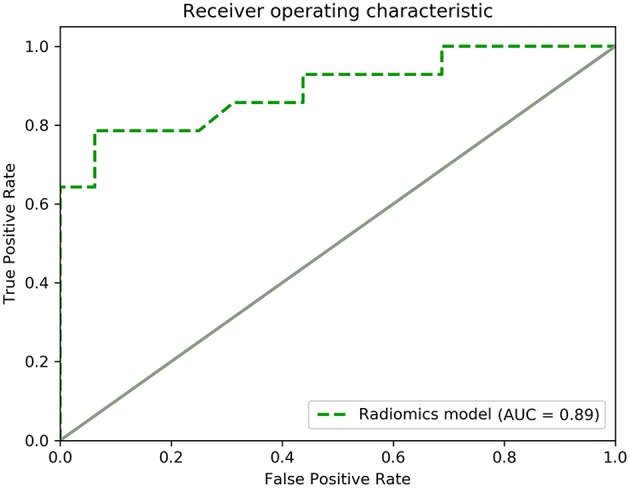
ROC curve for the performance of the radiomics model in the multicenter prospective validation.

Notably, the radiomics model performed well in the multicenter prospective validation for prediction of the tumor consistency, with an AUC and accuracy of 0.89 and 86.7%, respectively (Figure 8). These findings revealed the ability of the radiomics model to classify the tumor consistency in patients with acromegaly.

## Discussion

According to the guidelines, the initial cure rate of transsphenoidal surgery for macroadenomas is 40–50% when performed by experienced pituitary surgeons (8). In patients with acromegaly, a small residual tumor will postoperatively induce persistent GH hypersecretion (29). Therefore, determination of the tumor consistency before surgery is important to plan the surgical approach, avoid the need for a multistage surgical procedure, avoid the development of persistent acromegaly, and improve the surgical cure rate (30). Nonetheless, whether the tumor consistency can be predicted using MR imaging techniques remains unclear.

Several studies have been performed in an effort to predict the PA consistency using MR imaging techniques (11, 13, 22, 31–39); however, the ability of MR pituitary images to predict the PA consistency is controversial (22). In some studies, the apparent diffusion coefficient was not markedly correlated with the PA consistency on diffusion-weighted imaging (35), enhanced reconstructed diffusion-weighted imaging (36), or line-scan diffusion-weighted imaging sequences (37). Bahuleyan et al. (22) suggested that the MR imaging signal intensity alone could not accurately predict the consistency of pituitary macroadenomas. Some other scholars have indicated that contrast-enhanced fast imaging employing steady-state acquisition (11), dynamic MR imaging acquisition (31), and MR elastography (32) sequences can potentially offer preoperative information regarding the PA consistency. In addition, Smith et al. (34) demonstrated a statistically significant difference in the T2WI intensity ratio of adenoma to cerebellar peduncle between soft and firm PAs. On this account, neurosurgeons can effectively prepare for surgical procedures. Moreover, our findings suggest numerous limitations of those studies, such as poor prediction accuracy, small sample sizes, lack of internal, and multicenter validation, and complicated imaging sequences (Table 5). As a result, the development of a highly effective and widely applicable method for preoperative prediction is urgently needed.

**Table 5:** Summary of the method and outcome of previous PA consistency prediction studies.

**References**	**Year**	**Patient number**	**MRI imaging sequence**	**Indicator**	**Clinical data combined**	**Internal validation**	**Multicenter validation**	**Prospective validation**	**Outcome**
Romano et al. (31)	2017	21	DCE-T1WI	SIR	No	No	No	No	AUC = 0.949
Hughes et al. (32)	2016	10	MRE	Signal intensities	No	No	No	No	Difference^#^
Ma et al. (33)	2016	48	CE T1-SE	SIR of tumor to normal frontal white matter	No	No	No	No	Difference^#^
Wei et al. (13)	2015	38	T2WI ADC imaging	Signal intensity	No	No	No	No	AUC: 0.52–0.79
Smith et al. (34)	2015	36	T2WI	SIR of adenoma to cerebellar peduncle	No	No	No	No	Difference^#^
Yamamoto et al. (11)	2014	29	CE 3D-FIESTA	Intratumoral hyperintense dots	No	No	No	No	Difference^#^
Alimohamadi et al. (35)	2014	30	DWI	ADC value	No	No	No	No	Inscrutability
Mahmoud et al. (36)	2011	24	CE-reconstruction DWI	ADC value	No	No	No	No	Inscrutability
Suzuki et al. (37)	2007	19	Line-scan DWI	ADC value	No	No	No	No	Inscrutability
Bahuleyan et al. (22)	2006	80	T2WI	Homogeneously hypointense	No	No	No	No	Inscrutability
Pierallini et al. (38)	2006	22	DWI and ADC imaging	SIR of tumor to white matter and ADC values	No	No	No	No	Difference^#^
Iuchi et al. (39)	1998	26	T2WI	Signal intensities and homogeneous enhancement	No	No	No	No	Difference^#^

*Difference^#^ means the imaging indicator differed between different tumor consistency groups. DCE, dynamic contrast enhanced; T1WI, T1-weighted imaging; T2WI, T2-weighted imaging; MRE, Magnetic resonance elastography; T1-SE, T1-spin echo; ADC, Apparent diffusion coefficient; CE, contrast enhanced; SIR, Signal Intensity Ratio*.

As an emerging study field, radiomics can possibly depict the intratumoral heterogeneity based on quantitative and classified high-throughput data (40). Radiomics is a new area of study in which quantitative and high-throughput data are extracted, processed, and analyzed to explore their relationships with valuable information (15, 41). The radiomics process first converts radiographic images into mineable data in four steps: image acquisition and reconstruction, segmentation of the ROI, feature extraction and quantification, and establishment of predictive and prognostic models. Novel image-based computational models have played increasingly important roles in accurate diagnosis and treatment guidance in the field of neuro-oncology thanks to the development of clinical imaging data (42). As suggested in numerous studies, radiomics analysis can be quite effective. Compared with soft PAs, firm PAs contain large amounts of collagen and are more homogeneously enhanced after injection of a gadolinium-DTPA contrast agent (39). The collagen content is the main factor affecting the texture of PAs and has an impact on the performance of MR imaging (13). Therefore, the use of radiomics technology to predict the tumor consistency in patients with acromegaly has a good theoretical basis and is promising.

A radiomics signature comprising four selected features (three texture features and one wavelet feature) was constructed in the present study. Importantly, the constructed radiomics signature was found to be able to successfully classify both soft and firm tumors in patients with acromegaly. Recent studies have indicated that textural features can serve as imaging markers to predict the PA subtype (43) and the glioma stage (44, 45). Textural features have also been depicted as patterns or spatial distributions of voxel intensity, and they can be computed from the GLCM (46). The voxel intensity values in the volume of interest are required to determine the representative texture matrix (47), and such a step can reduce the image noise and normalize the intensities among all patients. Thus, it is possible to directly compare all of the computed textural features among different patients. Table 3 suggests that the most relevant imaging feature is the GLCM. The GLCM, which is a type of texture-analysis method, can calculate the frequency of occurrence of pixel pairs with specific values at a specified spatial relationship in an image (16) as supported by several recent studies (16, 48). The GLCM can characterize the tumor heterogeneity; therefore, to distinguish between a soft and firm tumor, it is important to obtain the statistical measures and to create the radiomics signature based on the GLCM. The radiomics model constructed in this study incorporated both the radiomics signature and the selected clinical features, with an AUC of 0.83 (95% CI, 0.81–0.85) and 0.81 (95% CI, 0.78–0.83) in the primary and validation cohorts, respectively. More importantly, this radiomics model displayed good calibration and discrimination. Additionally, this model was convenient to use and could accurately predict the tumor consistency in a multicenter prospective validation before surgery.

To our knowledge, the current study is the first to evaluate the consistency of pituitary macroadenomas by means of a radiomics approach. Our study has certain advantages over retrospective studies (Table 5). First, the large sample size in this study provided reliable results. Second, because patients with acromegaly may benefit from preoperative treatment with somatostatin analogs (49, 50), and because somatostatin analogs may induce histological, fibrous, and consistency changes in PAs (51), we only selected patients without a history of preoperative treatment to ensure the accuracy of the predicted results. Third, the preoperative prediction model constructed based on radiomics features incorporated both clinical and quantitative imaging features that were not susceptible to any degradation; moreover, the model was of high effectiveness and extensive application. Finally, the patients were divided into independent primary and validation cohorts for internal validation, and multicenter prospective validation was also conducted thereafter.

This study also had some limitations. First, although this was a multicenter study, we only conducted a holistic prospective validation because some centers provided fewer patients. More prospective datasets are needed for independent center validation and verification of the robustness and repeatability of this radiomics model. Second, different classification methods are used for PA consistency in clinical application, including two groups (11, 22, 34, 39), three groups (13, 32, 35, 37, 38), and other methods (33, 51). We chose to divide the tumors into two groups, but different classification methods may lead to different models and prediction results. Third, although most recent studies (32, 34, 35, 37–39) used only clinical data (surgical records and videos) to differentiate and classify the PA consistency, the cellular type, fibrous characteristics, and reticulin content have a significant relationship with consistency and can help to determine the consistency of PAs (11, 13, 30, 31, 35, 38, 39). Therefore, clinical data and pathological data should be combined in further experiments to provide a more reliable basis for the classification of tumor consistency. Finally, radiomics may serve as a complementary tool to transcriptomics, genomics, and proteomics, all of which may be used in combination to construct a more accurate model for prediction.

In summary, the findings of this study suggest that a radiomics model constructed based on a radiomics signature combined with clinical features can improve the accuracy of predicting the tumor consistency in patients with acromegaly. The as-constructed multiparametric radiomics model was further validated internally and externally, showing robustness. Moreover, the radiomics model performed better than any single model and achieved results superior to those in previous studies. Most importantly, it can serve as an effective noninvasive approach to distinguish the tumor consistency and determine individualized therapeutic schemes for patients with acromegaly.

## Data Availability

The data used to support the findings of this study are available from the corresponding author upon request.

## Ethics Statement

This study was carried out in accordance with the recommendations of the Ethical Review Committee of Peking Union Medical College Hospital with written informed consent from all prospective subjects. The protocol was approved by the Institutional Review Board of Peking Union Medical College Hospital.

## Author Contributions

All authors provided contributions to the study conception and design, acquisition of data or analysis and interpretation of data, drafting of the article, or revising it critically for important intellectual content, and final approval of the version to be published. All authors analyzed and interpreted the data. YF, MH, and AM revised the manuscript for important intellectual content. RW and MF take final responsibility for this article.

### Conflict of Interest Statement

The authors declare that the research was conducted in the absence of any commercial or financial relationships that could be construed as a potential conflict of interest.
